# Identification of leaky gut-related markers as indicators of metabolic health in Dutch adults: The Nutrition Questionnaires plus (NQplus) study

**DOI:** 10.1371/journal.pone.0252936

**Published:** 2021-06-04

**Authors:** Hiroyuki Hoshiko, Edith J. M. Feskens, Els Oosterink, Renata M. C. Ariens, Jurriaan J. Mes, Nicole J. W. de Wit

**Affiliations:** 1 HE Center, Suntory MONOZUKURI Expert Ltd., Kyoto, Japan; 2 Human Nutrition and Health, Wageningen University, Wageningen, The Netherlands; 3 Wageningen Food and Biobased Research, Wageningen University & Research, Wageningen, The Netherlands; Medical University Innsbruck, AUSTRIA

## Abstract

**Background and aim:**

Chronic inflammation is a primary risk factor for chronic metabolic disease and may be triggered by a “leaky gut.” Several biomarkers have been recognized to indicate intestinal permeability (i.e., leaky gut) and bacterial translocation. Nonetheless, which of these biomarkers exhibit the highest correlation with metabolic health parameters remains unclear. Hence, this study aimed to explore the correlation between leaky gut-related markers and metabolic health.

**Methods:**

Based on waist circumference, plasma fasting glucose, plasma gamma-glutamyl transpeptidase (GGT), and plasma LDL cholesterol, two groups of 40 subjects with the most extreme metabolic health profiles were selected from the NQplus cohort study (n = 2048), which was previously conducted by the Wageningen University’s Division of Human Nutrition. Eight potential leaky gut-related markers were selected from the literature and measured in serum or EDTA plasma samples of these selected individuals. These samples were also obtained from the NQplus cohort study.

**Results:**

From the leaky gut markers, levels of zonulin, lipopolysaccharide-binding protein, soluble CD14, bactericidal/permeability-increasing protein, and peptidoglycan were significantly higher in individuals with unhealthy metabolic profiles (p<0.05). No differences in EndoCAb IgM, EndoCAb IgA, and EndoCAb IgG were observed between healthy and unhealthy individuals. Stepwise regression analysis revealed that zonulin was substantially associated with metabolic health parameters such as BMI, blood glucose, triglyceride, GGT, and C-reactive protein levels. C-reactive protein, an inflammation marker, showed the most pronounced association with zonulin.

**Conclusions:**

Biomarkers that link a leaky gut and subsequent bacterial translocation to metabolic health were identified in this study. Especially zonulin may aid in monitoring a leaky gut and detecting individuals at risk for developing chronic metabolic diseases.

## Introduction

An impairment in intestinal integrity (i.e., “leaky gut” or increased intestinal permeability) is currently hypothesized to result in the translocation of potentially harmful compounds (bacteria, toxins, such as lipopolysaccharides [LPS], and waste), compromised metabolic detoxification, and induction of pro-inflammatory signals throughout the body, potentially leading to chronic disease development. A previous study has recently reported the association of increased intestinal permeability with visceral adiposity and liver fat accumulation [1], both of which are closely related to other metabolic disorders, including insulin resistance and elevated low-density lipoprotein (LDL) cholesterol levels. This suggests that a “leaky gut” may play a direct or indirect role in developing metabolic disorders related to metabolic syndrome [2].

Several biomarkers have been shown to indicate bacterial translocation and permeability. A previous study reported increased circulatory levels of zonula occludens-1 (a marker of increased gut permeability) and LPS in patients with type 2 diabetes mellitus [3]. Furthermore, bacterial DNA [4], LPS [5], and peptidoglycan (PG) have been detected as bacterial components in the blood and may initiate an inflammatory response. Our immune system immediately recognizes bacterial invasion and initiates the neutralization of LPS toxicity. Serum IgG, IgA, and IgM endotoxin-core antibodies are known markers of endotoxin exposure [6]. Lipopolysaccharide-binding protein (LBP), an acute-phase protein mainly secreted by the liver, modulates the LPS-induced immune response; particularly, circulatory LBP levels are considerably increased in patients with type 2 diabetes mellitus, especially those with morbid obesity [7]. The bactericidal/permeability-increasing protein (BPI) produced by neutrophils has been reported to exhibit antimicrobial activity and has been identified as an LPS-neutralizing protein [8]. Soluble CD14 (sCD14) is considered a marker of monocyte activation. LPS bound to CD14 is transferred to the TLR4/MD2 complex, followed by the activation of the NF-kB signaling pathway and the production of inflammatory cytokines responsible for activating the innate immune system [9].

As described above, several biomarkers have been recognized to indicate intestinal permeability (i.e., leaky gut) and bacterial translocation. Nonetheless, which of these biomarkers exhibit the highest correlation with metabolic health parameters remains unclear. Hence, the present study aimed to identify and select the most accurate and valid leaky gut-related markers (LGMs) that could be measured in plasma/serum and correlated with metabolic health status in the Dutch adult population.

## Materials and methods

### Subjects

**Fig 1:**
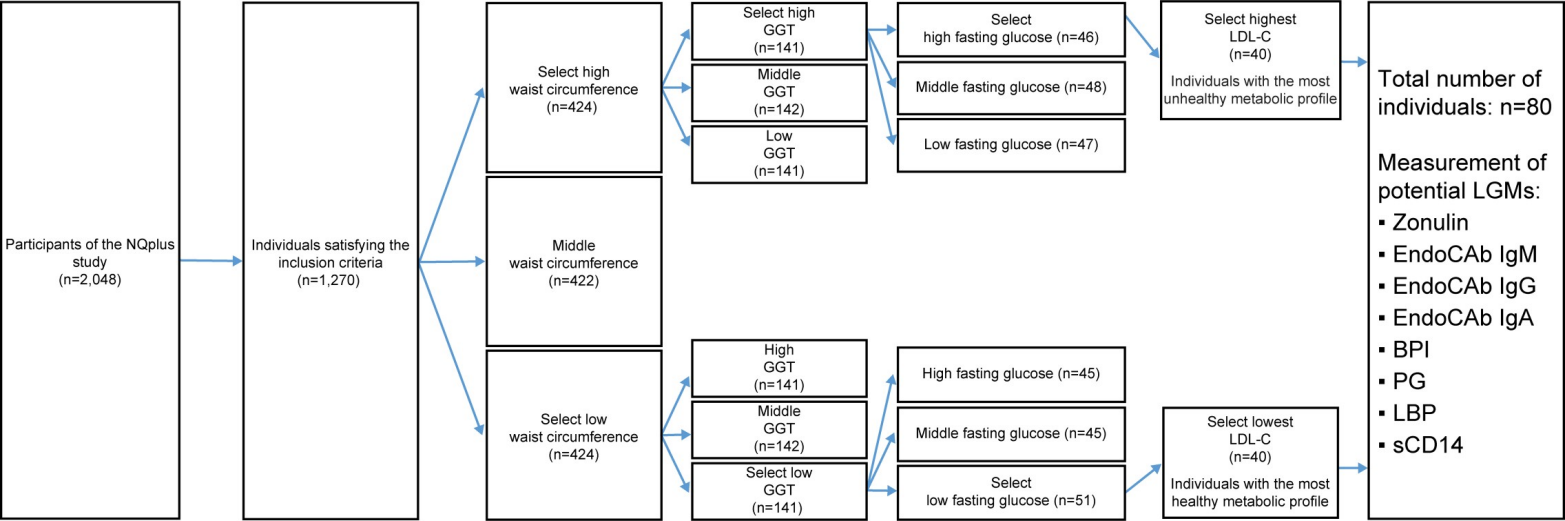
Schematic overview of subject selection from the NQplus study. Following this scheme, 40 individuals with the most healthy metabolic profiles and 40 individuals with the most unhealthy metabolic profiles from the NQplus study were selected.

Previously, the NQplus cohort study was conducted by the Wageningen University’s Division of Human Nutrition [10]. The NQplus study was approved by the Medical Ethical Committee of Wageningen University (NL34775.081.10) and was conducted according to the guidelines laid down in the Declaration of Helsinki. All participants gave written informed consent. This cohort study included 2,048 individuals from whom demographic data, food intake data, and metabolic health data were collected, including plasma and serum samples stored for future analyses. Using the data available from the NQplus cohort study, individuals with the most healthy (n = 40) and unhealthy (n = 40) metabolic profiles were now selected based on waist circumference, plasma fasting glucose level, plasma gamma-glutamyl transpeptidase level, and plasma LDL cholesterol level; the scheme illustrating subject selection is presented in Fig 1. In short, we will outline this strategy. We selected waist circumference as a first criterium as this has a strong association with metabolic health and other health risk indicators, such as blood glucose, triglycerides, HDL cholesterol [11]. Then GGT, a marker linked to liver pathologies (e.g., NAFLD, NASH) [12], was included in the selection procedure, as the leaky gut is previously also associated with these liver diseases [13, 14]. HDL cholesterol, triglycerides, and LDL cholesterol levels are traditional risk factors for cardiovascular disease. In our study, LDL cholesterol was included as a final marker for selection, mainly because it had a broader range (0.7–6.0 mmol/L) within the NQplus cohort population than HDL cholesterol (0.7–3.7 mml/L) and blood triglycerides (0.3–6.8 mmol/L), and more people exceeded the optimal LDL cholesterol levels of <2.6 mmol/L (~75%). We aimed for a more discriminative metabolic profile between the most metabolic healthy and unhealthy groups by including LDL cholesterol as a final marker. In every step of our selection procedure, we stratified for age (<54 years and ≥54y) and gender (male/female) to obtain ‘balanced’ groups for the most metabolic healthy and unhealthy subjects within the NQplus cohort population. In the final step, for each stratum (male < 54 years, male ≥ 54 years, female < 54 years, female ≥ 54 years), the 10 people with the lowest or highest LDL cholesterol levels were selected and allocated to the metabolic healthy or metabolic unhealthy group respectively. Current smokers, individuals with ethanol intake ≥60 g/day, and patients with a history of diabetes mellitus, myocardial infarction, heart failure, renal dysfunction, hepatic dysfunction (cirrhosis, hepatitis), and gastrointestinal disorders (stomach ulcer, ulcerative colitis, Crohn’s disease, celiac disease) were excluded from the selection procedure. From these 80 selected individuals, stored serum and EDTA plasma samples were now used for further analyses in the present study.

### Measurement of markers

Eight LGMs were selected based on their potential link to “leaky gut” and/or bacterial translocation. These LGMs were measured in the serum or EDTA plasma samples of the 80 individuals selected from the NQplus study. During the NQplus study, these samples were collected in the fasting state after a period of overnight fasting and were stored at −80°C in the Division of Human Nutrition, Wageningen University. Enzyme-linked immunosorbent assay was used to measure zonulin (K5601; Immundiagnostik AG, Bensheim, Germany) in serum as well as LBP (HK315-02; Hycult Biotech, Uden, Netherlands), EndoCAb IgM (HK504-IgM; Hycult Biotech), EndoCAb IgG (HK504-IgG; Hycult Biotech), EndoCAb IgA (HK504-IgA; Hycult Biotech), BPI (HK314-02; Hycult Biotech), sCD14 (HK320-02; Hycult Biotech), and PG (ABIN1116409; Antibodies-Online, Atlanta, GA, USA) in EDTA plasma according to the manufacturer’s protocol. Subsequent to LGMs, C-reactive protein (CRP), an inflammation marker, was measured in EDTA plasma (CRP range: 0.25–16 ng/mL) (EC1001-1; Assaypro LLC, St. Charles, MO, USA). All samples were analyzed in duplicate. For each ELISA, intra-assay CV was calculated as the average %CV of all samples measured in duplicate. The inter-assay CV was calculated as the average of the standard curve average %CV of plate 1 and plate 2. This resulted in the following values: zonulin (intra-assay CV 2.1%, inter-assay CV 2.1%), LBP (intra-assay CV 2.1%, inter-assay CV 2.4%), EndoCAb IgM (intra-assay CV 1.7%, inter-assay CV 2.7%), EndoCAb IgG (intra-assay CV 3.0%, inter-assay CV 3.3%), EndoCAb IgA LBP (intra-assay CV 2.7%, inter-assay CV 3.7%), BPI (intra-assay CV 2.9%, inter-assay CV 5.8%), sCD14 (intra-assay CV 4.5%, inter-assay CV 2.1%), PG (intra-assay CV 4.1%, inter-assay CV 5.3%), CRP (intra-assay CV 3.1%, inter-assay CV 4.8%).

### Statistical analysis

Statistical analyses were conducted using SPSS software version 22 (IBM Corp., Armonk, NY, USA). Only values with a coefficient of variation of <20% in the enzyme-linked immunosorbent assay were regarded as adequate and were included in the statistical analyses for all markers. Furthermore, outliers were included (analyzed by boxplots with interquartile range) because no reasonable arguments were available to exclude these samples from further analyses. We verified that these outliers did not substantially affect the statistical outcomes, as we performed analyses with and without outliers. The Shapiro-Wilk test was used to evaluate the normal distribution of markers.

Significant differences for normal distributed raw data were calculated using the Independent T-test, and significant differences for non-normal distributed raw data were calculated using the Mann-Whitney U test (S1 Table). For stepwise regression analyses, values were log_10_-transformed for all markers, and “0” or “below detection limit” was adjusted to half of the lowest value that could be measured. The metabolic markers were then set as dependent variables and the leaky gut markers as fixed predictors (independent variables). To also explore interactions with age and gender, first centered values were calculated for the log-transformed LGM values and gender and age, after which interaction was calculated by log-centered LGM * centered gender or centered age. These interaction values were then also included in the stepwise regression analyses as independent variables.

## Results

### Subjects

Two groups with the most extreme metabolic health profiles (A total of 80 individuals with the most healthy (n = 40) and unhealthy (n = 40) metabolic profiles) were selected from the NQplus cohort study. As expected, all metabolic parameters were significantly different between individuals with healthy metabolic profiles and those with unhealthy metabolic profiles (Table 1).

**Table 1:** Metabolic characteristics (mean ± SE) of individuals with healthy (n = 40) and unhealthy (n = 40) metabolic profiles in the pilot study.

Metabolic parameters	Individuals with healthy metabolic profiles	Individuals with unhealthy metabolic profiles	p-value^a^
**Weight (kg)**	66.3 (1.4)	96.4 (2.5)	<0.001
**BMI (kg/m**^**2**^**)**	22.1 (0.3)	29.7 (0.6)	<0.001
**Waist circumference (cm)**	79.3 (1.1)	107.7 (1.6)	<0.001
**Body fat (%)**	22.1 (1.5)	37.9 (1.2)	<0.001
**Glucose (mmol/L)**	4.7 (0.04)	6.4 (0.08)	<0.001
**HbA1c (mmol/mol)**	34.0 (0.3)	38.8 (0.6)	<0.001
**Total cholesterol (mmol/L)**	4.9 (0.1)	5.7 (0.1)	<0.001
**HDL (mmol/L)**	1.8 (0.08)	1.3 (0.06)	<0.001
**LDL (mmol/L)**	2.8 (0.1)	3.5 (0.1)	<0.001
**Triglycerides (mmol/L)**	0.7 (0.04)	2.0 (0.2)	<0.001
**ALT (U/L)**	20.5 (1.0)	34.9 (2.7)	<0.001
**GGT (U/L)**	9.5 (0.4)	56.0 (8.7)	<0.001
**CRP (mg/mL)**	2.5 (0.5)	8.4 (1.7)	<0.001

^a^Significance by the Independent T-test or the Mann–Whitney U test depending on normal distribution of the raw data as evaluated using the Shapiro–Wilk test. Only waist circumference, body fat, HbA1c, and LDL cholesterol showed a normal distribution (p>0.05).

BMI: body mass index, ALT: alanine aminotransferase, GGT: gamma-glutamyl transpeptidase, HbA1c: glycated hemoglobin A1c, HDL: high-density lipoprotein, LDL: low-density lipoprotein, CRP: C-reactive protein.

### Measurement of LGMs

LGMs with a potential direct or indirect relation to impairment in intestinal integrity and/or bacterial translocation were selected and measured in serum or plasma samples obtained from all 80 individuals. Table 2 summarizes the differences between individuals with healthy and unhealthy metabolic profiles for all measured markers. Those with unhealthy metabolic profiles exhibited significantly higher zonulin, LBP, sCD14, PG, and BPI levels. Data on metabolic characteristics and LGM levels are provided separately for sex and age (<54 and ≥54 years) in S2 and S3 Tables.

**Table 2:** LGM levels (mean ± SE) in individuals with healthy (n = 40) and unhealthy (n = 40) metabolic profiles in the pilot study.

LGMs	Individuals with healthy metabolic profiles	Individuals with unhealthy metabolic profiles	p-value^a^
**Zonulin (ng/mL)**	37.8 (1.1)	48.8 (1.7)	<0.001
**EndoCAb IgA (AMU/mL)**	49.0 (14.0)	63.6 (15.9)	0.27
**EndoCAb IgM (MMU/mL)**	1.8 (0.07)	1.8 (0.04)	0.26
**EndoCAb IgG (GMU/mL)**	91.4 (11.5)	90.5 (11.4)	0.92
**BPI (ng/mL)**	2.3 (0.5)	6.1 (1.8)	0.02
**PG (ng/mL)**	1.3 (0.5)	2.6 (1.1)	0.04
**LBP (μg/mL)**	10.2 (0.7)	12.6 (0.7)	0.01
**sCD14 (μg/mL)**	1.2 (0.04)	1.4 (0.06)	0.01

^a^Significance by the Mann–Whitney U test (raw data were not normally distributed, as evaluated using the Shapiro–Wilk test).

BPI: bactericidal/permeability-increasing protein, LBP: LPS-binding protein, sCD14: soluble CD14, PG: peptidoglycan.

### Relationship between LGM and metabolic health

**Fig 2:**
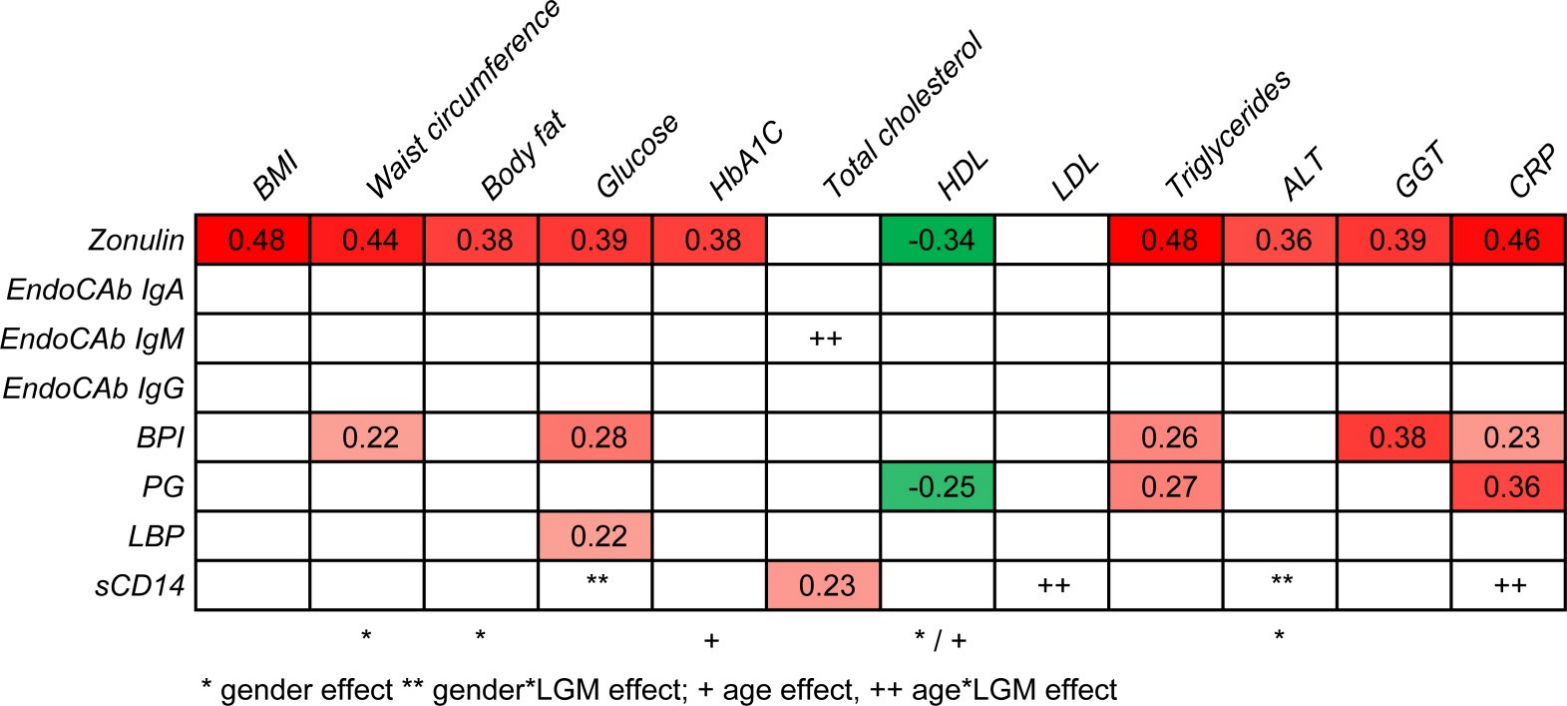
Stepwise linear regression analyses to test the relationship between LGM and metabolic markers. Standardized coefficient ß’s of the final model only reported if p<0.05. LGM were the independent variables, and the metabolic markers were the dependent variables. Red indicates a positive relationship (more intense color means a stronger relationship), green indicates a negative relationship.

To determine which LGMs are most closely associated with metabolic health, stepwise regression analysis was performed for each metabolic health marker (Fig 2). A significant main effect of gender was found for waist circumference, body fat, HbA1C, and ALT. As for HDL, the main effect was found for both gender and age. Among all LGMs, zonulin showed the most pronounced association with metabolic health, as a significant relationship was found with all metabolic markers, except total cholesterol and LDL cholesterol. BPI and PG both showed a link with triglycerides and CRP, where BPI was additionally associated with waist circumference, glucose and GGT, and PG showed a negative relationship with HDL cholesterol. LBP only showed a link with glucose and sCD14 with total cholesterol. For several metabolic markers, effects of sCD14 were found to interact with gender or age. Therefore, follow-up linear regression analyses were performed separately for males and females and/or for younger (<54y) and older people (≥54y). Results showed that the relationship between sCD14 and glucose was only significant in females (ß = 0.511, p = 0.001), and the relation between sCD14 and cholesterol was significant only in the younger age group (ß = 0.483, p = 0.001); by contrast, a significant relationship between sCD14 and CRP was only found in the older age group (ß = 0.359, p = 0.047).

Moreover, the effect of EndoCap IgM on total cholesterol was found to be significantly mediated by age, but follow-up analyses showed no significant relationship between EndoCap IgM and total cholesterol in either age group.

## Discussion

The present study investigated the relationship between potential “leaky gut” biomarkers and metabolic health. In individuals with the most unhealthy metabolic profiles, significantly higher (p<0.05) levels of zonulin, LBP, sCD14, BPI, and peptidoglycan were found.

For this study, two groups with the most extreme metabolic health profiles were selected from the NQplus cohort study (n = 2048 subjects), which seems to limit an accurate selection of most extreme metabolic healthy and unhealthy subjects on all metabolic health parameters. The average metabolic levels in individuals with the most unhealthy metabolic profiles did not meet the following criteria for metabolic syndrome: central obesity, defined as waist circumference ≥94 cm for Europid men and ≥80 cm for Europid women, with ethnicity-specific values for other groups; elevated triglyceride level (≥1.7 mmol/L); decreased HDL cholesterol level (<1.03 mmol/L in males and <1.29 mmol/L in women); and increased fasting plasma glucose level (≥5.6 mmol/L) [15]. This indicates that this group is not extremely metabolically unhealthy. Hence, the present study did not include morbidly obese individuals with a serious metabolic health problem. It would be interesting to investigate LGMs in the morbidly obese population to determine whether LGM levels are even more elevated in this extreme metabolic phenotype.

CRP was measured in this study to link LGMs to an inflammatory status, as low-grade inflammation is often associated with metabolic syndrome [16]. In our study, CRP levels were higher in individuals with unhealthy metabolic profiles than in those with healthy metabolic profiles, indicating that they are certainly at risk for developing metabolic syndrome. Stepwise regression analysis was performed for each metabolic health marker. A significant main effect of gender was found for some metabolic health markers, such as waist circumference, body fat, and HDL cholesterol. This is not surprising, as it is commonly known that there are substantial differences in waist circumference and body fat between men and women [17]. For waist circumference and HDL cholesterol, the diagnostic criteria for metabolic syndrome are even gender-dependent [15].

Among LGM, especially zonulin was substantially associated with metabolic health status, including a significant relationship with CRP. According to previous studies, these results show that zonulin levels were associated with the increased risk of overweight, obesity, and hyperlipidemia [18]. Furthermore, the potential link between zonulin and CRP is supported by the findings of van Hemert et al., who reported a reduction in both zonulin and CRP levels after an improvement in the intestinal barrier consequent to a probiotic intervention [19]. CRP also showed a significant relationship with BPI and PG and with sCD14 in the older age group. These data suggest that some LGMs can indeed be linked to an inflammatory status, as measured by CRP. sCD14 is linked to LPS, another serum marker that is linked to chronic inflammation in literature. Translocation of LPS from the intestine can occur through transcellular or paracellular pathways [20]. In this study, LPS quantification was unfortunately not evaluated. Although the Limulus amebocyte lysate assay is known to detect endotoxins, this assay is still not very accurate for serum or plasma samples owing to the presence of inhibitory factors. Some studies imply that sCD14 could be protective towards inflammatory signals via a blunting effect on LPS, and this potential function of sCD14 would be comparable to the neutralizing effect of HDL cholesterol on LPS [21]. In this study, sCD14 showed a link with blood glucose only in women. In the literature, there was an indication that women had higher levels of sCD14 than men, and the effect of estrogen on monocyte activation and TLR4 responses is proposed to be a causal factor for this [22]. These data support our finding that sCD14 showed sex-specific associations with metabolic health parameters.

We now studied the relationship between potential “leaky gut” biomarkers and metabolic health. Since this was a pilot study, we selected two subgroups within the NQplus cohort study with the most extreme metabolic health profiles. The primary objective of our study was to identify and select the most accurate and valid LGMs that could be measured in plasma/serum and are associated with metabolic health status. Further studies are necessary for a larger population to validate and/or strengthen the evidence for a relationship between intestinal integrity (leaky gut) and an unhealthy metabolic profile related to metabolic syndrome. Based on our current data, the biomarkers linking a leaky gut and subsequent bacterial translocation to metabolic health were identified. Zonulin was most substantially associated with metabolic health markers and CRP, which is a chronic inflammation marker. This biomarker is the most promising candidate to monitor a leaky gut and detecting individuals at risk for developing chronic metabolic diseases.

## Supporting information

S1 Table Anonymized data set. (XLSX)

S2 Table Metabolic characteristics and LGM levels separately for sex. (XLSX)

S3 Table Metabolic characteristics and LGM levels separately for age (<54 years and ≥54 years). (XLSX)
